# Pimavanserin for Parkinson's Disease psychosis: Effects stratified by baseline cognition and use of cognitive‐enhancing medications

**DOI:** 10.1002/mds.27488

**Published:** 2018-11-02

**Authors:** Alberto J. Espay, Michael T. Guskey, James C. Norton, Bruce Coate, Joaquin A. Vizcarra, Clive Ballard, Stewart A. Factor, Joseph H. Friedman, Anthony E. Lang, Niccole J. Larsen, Candace Andersson, Doral Fredericks, Daniel Weintraub

**Affiliations:** ^1^ Gardner Family Center for Parkinson's Disease and Movement Disorders, Department of Neurology University of Cincinnati Cincinnati Ohio USA; ^2^ ACADIA Pharmaceuticals Inc. San Diego California USA; ^3^ University of Exeter Medical School Exeter United Kingdom; ^4^ Jean and Paul Amos Parkinson's Disease and Movement Disorder Program, Department of Neurology Emory University School of Medicine Atlanta Georgia USA; ^5^ Department of Neurology Warren Alpert Medical School of Brown University Providence Rhode Island USA; ^6^ Movement Disorders Program, Butler Hospital Providence Rhode Island USA; ^7^ Edmond J. Safra Program in Parkinson's Disease and the Morton and Gloria Shulman Movement Disorders Clinic, Toronto Western Hospital University of Toronto Toronto Ontario Canada; ^8^ Departments of Psychiatry and Neurology Perelman School of Medicine at the University of Pennsylvania Philadelphia Pennsylvania USA; ^9^ Parkinson's Disease and Mental Illness Research, Education and Clinical Centers (PADRECC and MIRECC), Department of Veterans Affairs Philadelphia VA Medical Center Philadelphia Pennsylvania USA

**Keywords:** Parkinson's disease psychosis, pimavanserin, cognitive impairment, cognitive‐enhancing medications, cholinesterase inhibitors, memantine

## Abstract

**Background**: PD psychosis is often associated with cognitive impairment, including dementia, and involves dopaminergic, serotonergic, and cholinergic mechanisms.

**Objective**: To evaluate the differential effect of the antipsychotic pimavanserin, a selective serotonin 2A receptor inverse agonist, in PD psychosis patients with versus without cognitive impairment and in those receiving versus not receiving cognitive‐enhancing medications.

**Methods**: Data from the pivotal randomized clinical trial of pimavanserin for PD psychosis were stratified by (1) screening MMSE score as cognitively impaired (21‐24) versus unimpaired (≥25) and (2) concomitant use versus nonuse of cognitive‐enhancing medications. The primary outcome measure was change in the PD‐adapted Scale for the Assessment of Positive Symptoms.

**Results**: Mean (pimavanserin vs. placebo) change from baseline was larger in the cognitively impaired (n = 50; –6.62 vs. –0.91; *P* = 0.002) versus the cognitively unimpaired (n = 135; –5.50 vs. –3.23; *p* = 0.046) group. The comparable change was –6.04 versus –2.18 (*P* = 0.012) and –5.66 versus –3.15 (*P* = 0.041) in patients treated (n = 69) and not treated (n = 116) with concomitant cognitive‐enhancing medication. Pimavanserin was similarly tolerated across all cognitive groups with no additional safety concerns identified. Overall adverse event rates were comparable across the concomitant cognitive‐enhancing medication groups; however, rates of serious adverse events and discontinuations attributed to adverse events were increased in patients taking cholinesterase inhibitors.

**Conclusions**: The antipsychotic effect of pimavanserin is robust in PD patients with cognitive impairment and may be enhanced by concomitant cognitive‐enhancing medication use. Future prospective studies are needed to confirm these preliminary findings. © 2018 The Authors. *Movement Disorders* published by Wiley Periodicals, Inc. on behalf of International Parkinson and Movement Disorder Society.

Parkinson's disease (PD) psychosis (PDP), characterized by hallucinations and/or delusions, is a leading cause of disability and nursing home placement for PD patients and impacts quality of life for both patients and their caregivers.^1^, ^2^, ^3^ PDP has a lifetime prevalence of up to 60% among PD patients.^3^, ^4^ Risk factors include older age, greater disease severity, affective disorder, greater autonomic symptom burden,, disorders of sleep and wakefulness, including REM sleep behavior disorder, and cognitive impairment.^5^, ^6^, ^7^ In addition to psychosis, cognitive impairment, including dementia, is another common and disabling nonmotor symptom in PD.^8^, ^9^, ^10^, ^11^, ^12^ Numerous studies have reported a strong association between cognitive impairment and PDP.^8^, ^9^, ^11^, ^13^ Thus, it is important that an antipsychotic used to treat PDP be effective, well tolerated, and safe across the cognitive spectrum and when used in combination with cognitive‐enhancing medications.

Pimavanserin, a selective 5‐HT_2A_ inverse agonist, has demonstrated efficacy in a randomized, placebo‐controlled trial and is the only antipsychotic specifically approved in the United States to treat hallucinations and delusions associated with PDP.^14^ In addition to serotonergic dysregulation,^15^, ^16^ there is evidence that dopaminergic and cholinergic mechanisms are involved in the etiology of PDP.^6^ For example, dopaminergic medications, especially dopamine agonists, can induce or worsen hallucinations.^17^ Similarly, psychosis can be precipitated by anticholinergic medications.^18^ In addition, PDP is magnified in the setting of dementia (in part, a cholinergic‐deficiency state in PD),^19^ and there is preliminary evidence that cognitive‐enhancing procholinergic medications may also have antipsychotic effects.^20^, ^21^, ^22^

All antipsychotics approved by the U.S. Food and Drug Administration include a boxed warning for an increased risk of death in elderly patients with dementia‐related psychosis.^23^ Although in the pivotal pimavanserin trial a formal diagnosis of dementia was exclusionary,^14^ it allowed a Mini‐Mental State Examination (MMSE)^24^ score of ≥ 21, thus likely including a subset of patients with either mild cognitive impairment or mild dementia. We hypothesized that pimavanserin would have similar efficacy and tolerability in cognitively impaired PDP patients compared to cognitively unimpaired patients, and that PDP patients treated concomitantly with pimavanserin and a cognitive‐enhancing medication would experience greater antipsychotic benefit than those taking pimavanserin alone.

## Patients and Methods

For this study, we used data from a 6‐week randomized, double‐blind, placebo‐controlled, phase 3 trial of pimavanserin (PIM) 34 mg (equivalent to 40 mg of pimavanserin tartrate) in patients with PDP (ClinicalTrials.gov NCT01174004; Study ACP‐013‐020).^14^ In brief, adults aged ≥40 years and satisfying diagnostic criteria for PDP,^25^ with symptoms of psychosis present for at least 1 month and severe enough to require antipsychotic drug treatment, were randomized (1:1) to receive either PIM 34 mg daily or placebo. Patients were required to have an MMSE score ≥21 at screening. The primary outcome was the change in the 9‐item Scale for the Assessment of Positive Symptoms adapted for PD (SAPS‐PD; scores 0‐45, higher scores indicating greater severity of psychosis).^26^ Although the original SAPS^27^ was designed for use in schizophrenia, it is one of only four scales recommended by the International Parkinson and Movement Disorder (MDS) Society Task Force on Rating Scales in PD for the assessment of response of psychosis to new treatments.^28^ Studies using the SAPS in clinical trials of PDP have shown that it is sensitive to change in response to treatment.^29^, ^30^, ^31^ The SAPS‐PD is a shortened version that contains only items relevant to PDP while retaining the reliability and sensitivity to change of the larger scale.^26^ In a previous analysis, a 1‐unit change in clinical global impression correlated to a 2.33‐point change in SAPS‐PD, a magnitude reflecting the minimal clinically important change in response to treatment.^26^

For this analysis, patients were stratified by MMSE score into cognitively impaired (21‐24; N = 50) and cognitively unimpaired (≥25; N = 135). The cutoff utilized for this analysis is slightly more stringent than the MMSE score ≤25 recommended by the MDS Taskforce on Dementia in PD to screen for dementia.^32^ The primary outcome was the change in SAPS‐PD score at day 43,^14^, ^26^ and secondary outcomes were Clinical Global Impression–Improvement (CGI‐I) score and tolerability. We also assessed efficacy and tolerability based on the concomitant use of cognitive‐enhancing medications (i.e., either acetylcholinesterase inhibitors [ChI] or memantine [Mem]).

### Statistical Analysis

The safety analysis set included all patients who received at least 1 dose of study medication. The efficacy analysis set included all patients who had at least one postbaseline SAPS‐PD assessment (modified intent‐to‐treat). The SAPS‐PD change from baseline was analyzed with the mixed‐model repeated measures (MMRM) method. The model included fixed categorical effects of treatment (PIM or placebo), visit (days 15, 29, or 43), and treatment‐by‐visit interaction and the continuous fixed covariate of baseline score. Missing values were not imputed. CGI‐I analyses evaluated observed cases only. Significance (α < 0.05) was determined based on the two‐sided *P* value for treatment difference at specified visits from MMRM analysis. To evaluate the treatment effect over placebo between the subgroups with and without cognitive impairment or with and without cognitive‐enhancing medications, an additional MMRM was performed using the aforementioned model with an additional subgroup factor and its associated two‐ and three‐way interaction terms. All statistical analyses we performed using SAS/STAT software (version 9.4 for Windows Server 2012; SAS Institute Inc., Cary, NC).

## Results

The overall efficacy population included 185 participants: 95 in the PIM group and 90 in the placebo group. The safety population included 198 participants: 104 in the PIM group and 94 in the placebo group.

### Baseline Demographics

Age, sex, PD duration, and duration of psychosis were comparable across the cognitive groups and regardless of coadministered cognitive‐enhancing medication (Table 1). For the entire cohort, participants were in their early seventies with an MMSE score of ∼26. Among PIM‐treated patients, those in the cognitively impaired group compared with the overall study population were less likely to be male, had a longer duration of PD, and had a higher baseline SAPS‐PD score, but the differences were not statistically significant. A total of 69 subjects (37%) were taking cognitive‐enhancing medications, with 12 patients taking more than one. There was a lower proportion of women on Mem in the placebo group. Because there was no correlation between MMSE scores and cognitive‐enhancing medication usage (Supporting Information Fig. S1; r_pb_ = –0.0751; *P* = 0.309), an analysis of the use of cognitive‐enhancing medication use stratified by MMSE was not performed.

**Table 1:** Baseline demographic and clinical features

	Efficacy Analysis Set		MMSE 21 to 24		MMSE ≥ 25		Any Cognitive‐Enhancing Medication		Any Acetylcholinesterase Inhibitor		Memantine	
	PIM	Placebo	PIM	Placebo	PIM	Placebo	PIM	Placebo	PIM	Placebo	PIM	Placebo
Demographics	(N = 95)	(N = 90)	(n = 29)	(n = 21)	(n = 66)	(n = 69)	(N = 33)	(N = 36)	(N = 31)	(N = 32)	(N = 6)	(N = 12)
Age (years)	72.4 (6.6)	72.4 (7.9)	73.4 (6.4)	72.5 (9.1)	72.0 (6.6)	72.3 (7.6)	73.1 (6.10)	72.9 (8.4)	72.9 (6.0)	74.1 (7.9)	73.2 (9.2)	72.5 (9.2)
Sex (%), male	64 (67.4)	52 (57.8)	18 (62.1)	16 (76.2)	46 (69.7)	36 (52.2)	21 (63.6)	27 (75.0)	20 (64.5)	25 (78.1)	3 (50.0)	10 (83.3)
PD duration (years)	9.7 (6.5)	10.6 (6.7)	11.1 (6.9)	12.0 (7.2)	9.0 (6.3)	10.2 (6.5)	10.7 (7.7)	8.4 (5.0)	11.0 (7.8)	8.9 (5.0)	9.8 (7.3)	9.0 (4.1)
PDP duration (months)	30.9 (30.0)	36.4 (39.6)	30.8 (36.1)	34.2 (21.8)	30.9 (27.3)	37.1 (43.7)	37.8 (36.0)	32.6 (26.8)	39.7 (36.3)	33.1 (27.6)	29.2 (21.6)	31.3 (17.0)
SAPS‐PD	15.9 (6.1)	14.7 (5.6)	16.9 (6.0)	14.2 (5.1)	15.5 (6.2)	14.9 (5.7)	16.8 (6.5)	14.6 (5.9)	16.8 (6.6)	15.1 (5.9)	18.0 (6.3)	12.1 (2.8)
CGI severity	4.3 (0.9)	4.3 (0.9)	4.5 (0.9)	4.8 (0.8)	4.2 (0.9)	4.2 (0.9)	4.0 (0.8)	4.5 (0.9)	4.0 (0.8)	4.6 (1.0)	4.2 (0.8)	4.5 (1.0)
MMSE	26.0 (2.6)	26.6 (2.4)	22.8 (1.1)	23.1 (1.1)	27.4 (1.6)	27.6 (1.5)	25.9 (2.4)	26.1 (2.6)	26.0 (2.5)	25.9 (2.7)	25.2 (1.9)	26.4 (2.35)

Mean (SD).

### SAPS‐PD Changes in Cognitively Impaired Versus Cognitively Unimpaired

**Figure 1:**
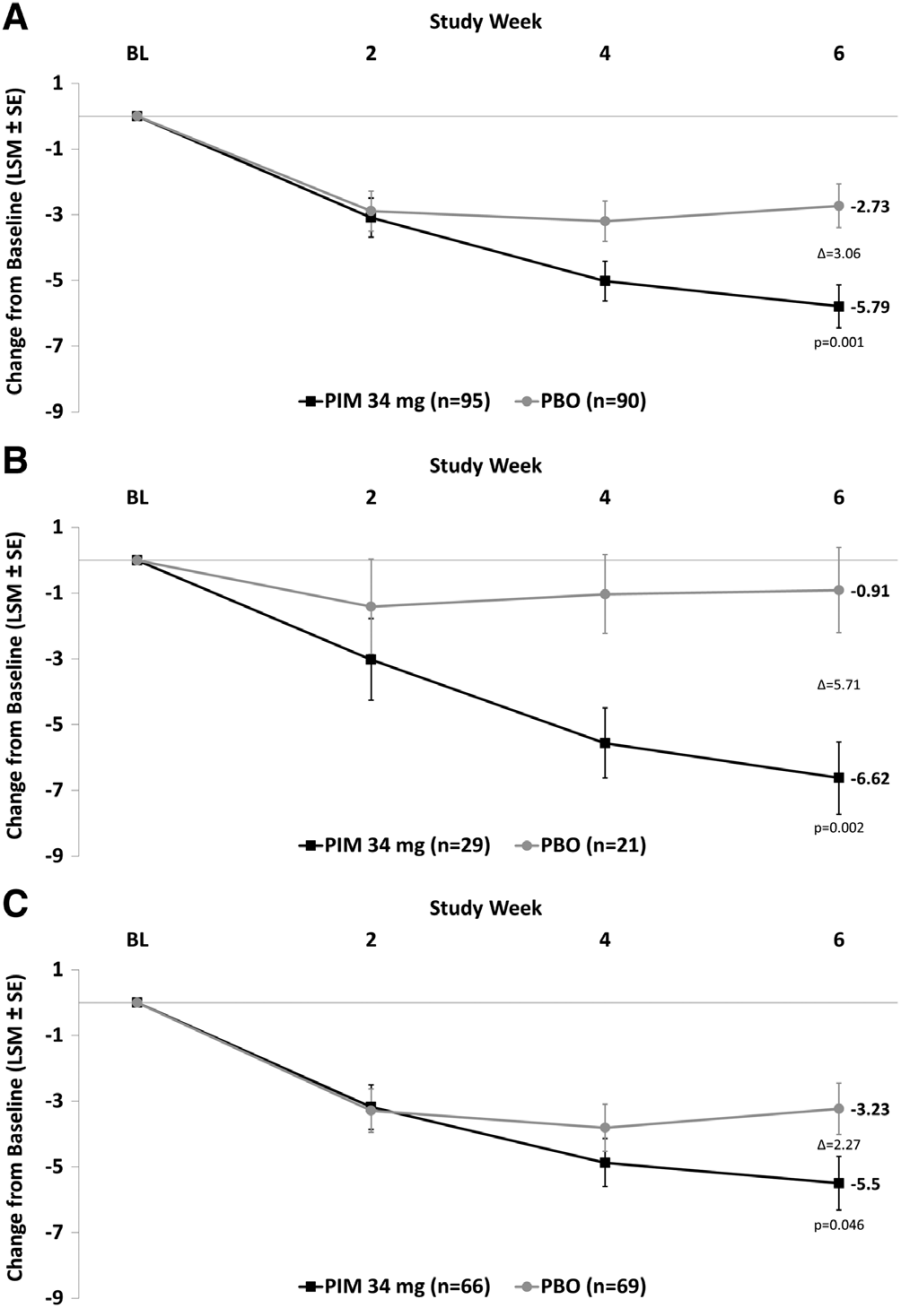
SAPS‐PD change from baseline stratified by baseline MMSE score. SAPS‐PD changes in: (A) overall population, (B) MMSE 21 to 24, and (C) MMSE ≥ 25. LSM, least squares mean; SE, standard error; PBO, placebo.

In the overall study population, the mean difference in SAPS‐PD score change from baseline for PIM versus placebo was –3.06 at day 43 (*P* = 0.001; Fig. 1A). PIM was superior to placebo in both the cognitively impaired and normal cognition subgroups. The least squares (LS) mean change from baseline in SAPS‐PD scores at day 43 in the cognitively impaired group was –6.62 with PIM versus –0.91 with placebo (PIM minus placebo = –5.71; 95% confidence interval [CI]: –9.17 to –2.24; *P* = 0.002; Fig. 1B). In the cognitively unimpaired group, the LS mean change in SAPS‐PD from baseline to day 43 was –5.50 with PIM versus –3.23 with placebo (PIM minus placebo = –2.27; 95% CI: –4.50 to –0.04; *p* = 0.046; Fig. 1C). The between‐group difference in SAPS‐PD score change from baseline for those with and without cognitive impairment was not statistically significant.

### CGI‐I in Cognitively Impaired Versus Cognitively Unimpaired

**Figure 2:**
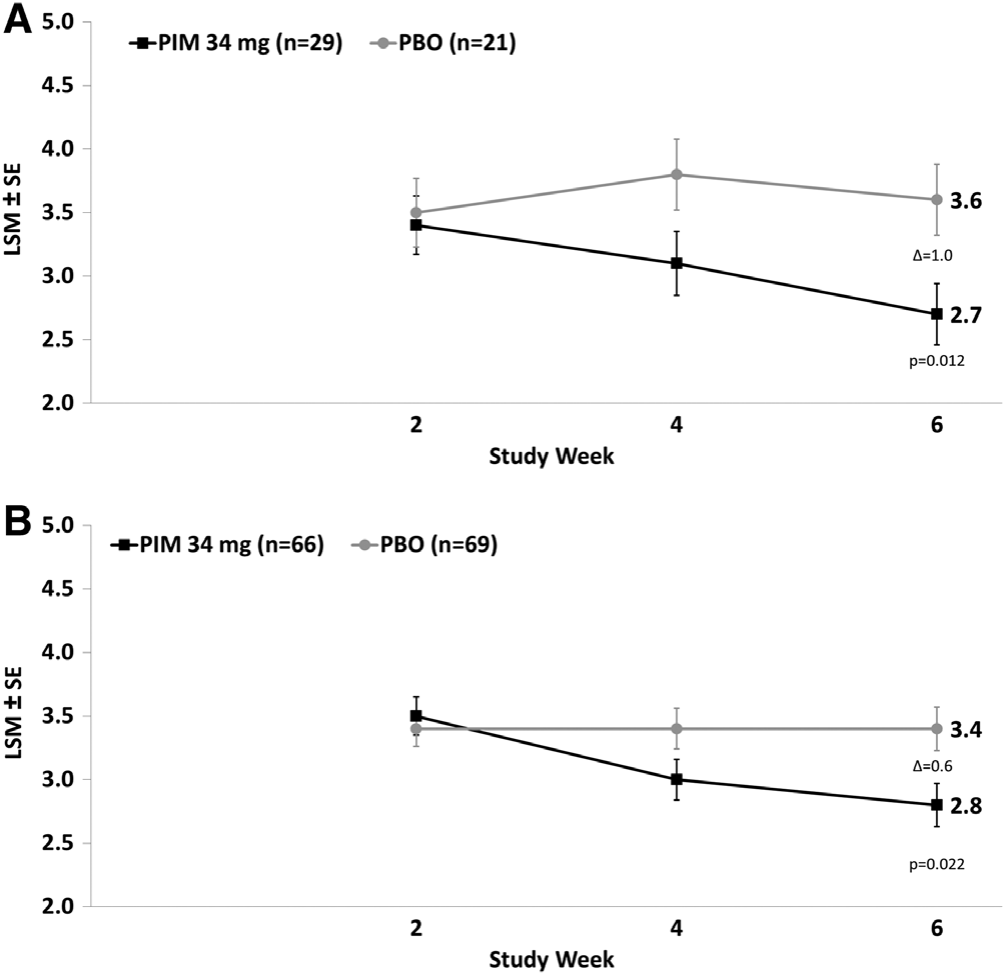
CGI‐I score in: (A) MMSE 21 to 24, and (B) MMSE ≥ 25. LSM, least squares mean; SE, standard error; PBO, placebo.

Among patients with cognitive impairment, the mean difference in CGI‐I score at day 43 for pimavanserin versus placebo was –1.0 (*P* = 0.012), and for the cognitively unimpaired group the mean difference for pimavanserin versus placebo was –0.6 (*p* = 0.022; Fig. 2). The between‐group difference in CGI‐I for those with and without cognitive impairment was not statistically significant.

### SAPS‐PD Changes With Concomitant Cognitive‐Enhancing Medications

**Figure 3:**
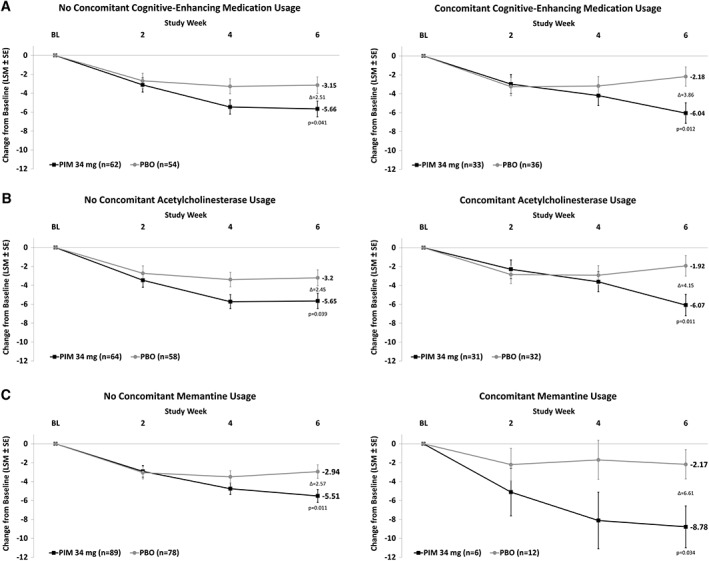
SAPS‐PD change from baseline in patients with versus without cognitive‐enhancing medications. The change in SAPS‐PD in subjects taking PIM or placebo (PBO), with or without concomitant cognitive‐enhancing medication (A), any cholinesterase inhibitor (B), and memantine (C). LSM, least squares mean; SE, standard error.

In the concomitant cognitive‐enhancing medication group, the LS mean change from baseline in SAPS‐PD scores at day 43 was –6.04 for PIM + cognitive‐enhancing medication versus –2.18 for placebo + cognitive‐enhancing medication, a difference of –3.86 (*P* = 0.012), compared to –5.66 and –3.15 in the PIM and placebo groups not taking cognitive‐enhancing medication, a difference of –2.51 (*p* = 0.041; Fig. 3A). The between‐group difference in SAPS‐PD score change from baseline for those treated with versus without concomitant cognitive‐enhancing medication was not statistically significant. Similarly, there was also a greater change from baseline in SAPS‐PD for patients taking PIM with ChI or with Mem when compared to those not taking these concomitant medications (Fig. 3B,C).

### CGI‐I With Concomitant Cognitive‐Enhancing Medications

Among patients taking concomitant cognitive‐enhancing medications, the mean difference in CGI‐I score at day 43 for pimavanserin versus placebo was –0.8 (*P* = 0.01); for patients not taking them, the mean difference was –0.6 (*P* = 0.03; Supporting Information Fig. S2). The between‐group difference in improvement in CGI‐I for those treated with versus without concomitant cognitive‐enhancing medication was not statistically significant. Greater improvements in CGI‐I were also noted for patients taking PIM with ChI or with MEM when compared to those not taking these concomitant medications.

### Tolerability and Safety

The incidence of adverse events (AEs) leading to discontinuation was numerically higher in PIM versus placebo across all cognitively defined groups: full population (9.6% vs. 3.2%); cognitive impairment (9.4% vs. 8.7%); and normal cognition (9.9% vs. 1.4%; Table 2). The most common AEs, occurring in >5% of PIM‐treated patients overall, were urinary tract infection, fall, peripheral edema, hallucinations, nausea, and confusional state. The latter was reported more often in the pimavanserin group across all three groups: full population (5.8% vs. 3.2%); cognitive impairment (6.3% vs. 0.0%); and cognitively unimpaired (5.6% vs. 4.2%). Falls were reported more often in the PIM: full population (10.6% vs. 8.5%); cognitively impaired (12.5% vs. 8.7%); and cognitively unimpaired (9.9% vs. 8.5%).

**Table 2:** Incidence (%) of AEs based on baseline MMSE and concomitant cognitive‐enhancing medication

	Safety Analysis Set		MMSE 21 to 24		MMSE ≥ 25		Any Cognitive‐Enhancing Medication		Any Acetylcholinesterase Inhibitor		Memantine		No Cognitive‐Enhancing Medication	
	PIM	Placebo	PIM	Placebo	PIM	Placebo	PIM	Placebo	PIM	Placebo	PIM	Placebo	PIM	Placebo
	(N = 104)	(N = 94)	(n = 32)	(n = 23)	(n = 71)	(n = 71)	(N = 39)	(N = 39)	(N = 37)	(N = 35)	(N = 8)	(N = 13)	(N = 65)	(N = 55)
Most common AEs^a^														
Urinary tract infection	14 (13.5)	11 (11.7)	2 (6.3)	2 (8.7)	12 (16.9)	9 (12.7)	6 (15.4)	5 (12.8)	6 (16.2)	3 (8.6)	—	3 (23.1)	8 (12.3)	6 (10.9)
Fall	11 (10.6)	8 (8.5)	4 (12.5)	2 (8.7)	7 (9.9)	6 (8.5)	8 (20.5)	3 (7.7)	8 (21.6)	3 (8.6)	1 (12.5)	—	3 (4.6)	5 (9.1)
Peripheral edema	7 (6.7)	3 (3.2)	2 (6.3)	—	5 (7.0)	3 (4.2)	1 (2.6)	1 (2.6)	1 (2.7)	1 (2.9)	—	—	6 (9.2)	2 (3.6)
Hallucination	7 (6.7)	1 (1.1)	1 (3.1)	—	6 (8.5)	1 (1.4)	4 (10.3)	1 (2.6)	4 (10.8)	1 (2.9)	1 (12.5)	—	3 (4.6)	—
Nausea	6 (5.8)	6 (6.4)	3 (9.4)	4 (17.4)	3 (4.2)	2 (2.8)	3 (7.7)	4 (10.3)	2 (5.4)	4 (11.4)	1 (12.5)	1 (7.7)	3 (4.6)	2 (3.6)
Confusional state	6 (5.8)	3 (3.2)	2 (6.3)	—	4 (5.6)	3 (4.2)	2 (5.1)	1 (2.6)	2 (5.4)	1 (2.9)	—	—	4 (6.2)	2 (3.6)
Insomnia	5 (4.8)	4 (4.3)	2 (6.3)	1 (4.3)	3 (4.2)	3 (4.2)	2 (5.1)	2 (5.1)	1 (2.7)	2 (5.7)	1 (12.5)	1 (7.7)	3 (4.6)	2 (3.6)
Constipation	4 (3.8)	2 (2.1)	1 (3.1)	—	3 (4.2)	2 (2.8)	2 (5.1)	1 (2.6)	2 (5.4)	—	—	1 (7.7)	2 (3.1)	1 (1.8)
Arthralgia	3 (2.9)	2 (2.1)	—	1 (4.3)	3 (4.2)	1 (1.4)	2 (5.1)	1 (2.6)	2 (5.4)	1 (2.9)	—	—	1 (1.5)	1 (1.8)
Psychotic disorder	3 (2.9)	2 (2.1)	2 (6.3)	2 (8.7)	1 (1.4)	—	2 (5.1)	1 (2.6)	2 (5.4)	1 (2.9)	—	—	1 (1.5)	1 (1.8)
Back pain	3 (2.9)	1 (1.1)	—	—	3 (4.2)	1 (1.4)	1 (2.6)	—	1 (2.7)	—	—	—	2 (3.1)	1 (1.8)
Contusion	3 (2.9)	1 (1.1)	3 (9.4)	1 (4.3)	—	—	2 (5.1)	1 (2.6)	2 (5.4)	1 (2.9)	—	—	1 (1.5)	—
Diarrhea	3 (2.9)	1 (1.1)	1 (3.1)	—	2 (2.8)	1 (1.4)	3 (7.7)	1 (2.6)	2 (5.4)	—	1 (12.5)	1 (7.7)	—	—
Dehydration	3 (2.9)	—	1 (3.1)	—	2 (2.8)	—	2 (5.1)	—	2 (5.4)	—	—	—	1 (1.5)	—
Any AE	74 (71.2)	59 (62.8)	22 (68.8)	12 (52.2)	52 (73.2)	47 (66.2)	28 (71.8)	24 (61.5)	26 (70.3)	20 (57.1)	4 (50.0)	8 (61.5)	46 (70.8)	35 (63.6)
Any serious AE	11 (10.6)	4 (4.3)	3 (9.4)	2 (8.7)	8 (11.3)	2 (2.8)	6 (15.4)	2 (5.1)	6 (16.2)	2 (5.7)	1 (12.5)	1 (7.7)	5 (7.7)	2 (3.6)
AE leading to d/c	10 (9.6)	3 (3.2)	3 (9.4)	2 (8.7)	7 (9.9)	1 (1.4)	6 (15.4)	2 (5.1)	6 (16.2)	2 (5.7)	1 (12.5)	—	4 (6.2)	1 (1.8)
Fatal AE	2 (1.9)	1 (1.1)	1 (3.1)	1 (4.3)	1 (1.4)	—	2 (5.1)	1 (2.6)	2 (5.4)	1 (2.9)	—	—	—	—

a AEs occurring in > 2 of the PIM‐treated patients in the overall safety analysis group (1 patient did not have an MMSE score so could not be categorized for the MMSE analysis).

AE rates were comparable across the concomitant cognitive‐enhancing medication groups; however, rates of serious AEs and discontinuations because of AEs were increased in patients taking a ChI (Table 2). The rate of study discontinuation because of AEs was 16.2% in the PIM + ChI group, 12.5% in the PIM + Mem, and 6.2% in the PIM‐without concomitant cognitive‐enhancing medication. The corresponding values in the placebo groups were 5.7%, 0.0%, and 1.8%. The mean (standard deviation; SD) heart rate‐corrected QT interval (QTcF) change from baseline to the last assessment in PIM + ChI was + 8.2 ms (23.5) ms, compared to + 5.3 (20.5) ms in PIM without ChI, and +4.7 (18.2) ms in PIM+Mem, compared to + 6.4 (21.8) ms in PIM without Mem. The corresponding values in the placebo groups were –0.6 (18.5) ms, + 0.3 (16.3) ms, –1.2 (10.1) ms, and +0.2 (17.9) ms. There was no observed QTcF >500 ms in any group.

With PIM treatment, one death each occurred in the cognitively impaired and normal cognition subgroups; in the placebo group, one death occurred in the cognitively impaired subgroup. All deaths were regarded as unrelated to study drug.^14^

## Discussion

In this subgroup analysis, pimavanserin demonstrated significant improvement in SAPS‐PD and CGI‐I scores among patients with PDP regardless of baseline cognition, but with larger responses in patients with impaired cognitive performance at baseline. Additionally, in participants taking concomitant cognitive‐enhancing medications, there was also a larger numerical SAPS‐PD effect compared to those not taking cognitive‐enhancing medications, although not statistically significant given the low power of this post‐hoc analysis. AEs were similar across the subgroups based on MMSE scores, but discontinuations because of an AE were more common in patients taking cognitive‐enhancing medications.

Although psychotic symptoms in patients with PDP have a marked impact on patients and their caregivers,^33^, ^34^, ^35^ few treatment options are both safe and effective. Atypical antipsychotic drugs are commonly used for treating PDP, but have been associated with increased morbidity.^36^ Furthermore, aside from clozapine and PIM,^29^, ^37^ no other agents have been deemed efficacious by the MDS.^38^ To compound the difficulty, PDP is often associated with comorbid dementia, for which all antipsychotics have a boxed warning.^23^ Furthermore, there are very limited data evaluating the safety and efficacy of antipsychotics in PDP patients with dementia compared to PDP patients without dementia. The only published study of which we are aware is a retrospective chart review of patient responses to quetiapine, which included 20 PDP patients with dementia and 19 without, based on diagnostic criteria from the *Diagnostic and Statistical Manual of Mental Disorders*, revised third edition. In this study, improvement in psychosis was reported in a similar proportion of patients, but a greater number of demented patients experienced a worsening of motor symptoms.^39^ Our analysis suggests that PIM may provide a safe and efficacious treatment option for PDP patients with cognitive impairment, as well as those on cognitive‐enhancing medications.

Further studies of PIM in PDP with a broader range of cognitive abilities (i.e., normal cognition, cognitive impairment, and dementia) are needed to replicate and further evaluate these findings of efficacy, tolerability, and safety in PD patients with cognitive impairment. Because previous studies have suggested antipsychotic effects for ChI and Mem,^22^, ^40^, ^41^ future studies should include stratification by ChI and Mem use to ensure a thorough analysis of the effects of these medications on psychosis. A recent study for PIM in patients with Alzheimer's disease psychosis showed benefit,^42^ and another study of PIM for psychosis in multiple dementing disorders is ongoing (NCT03325556).

In addition to the post‐hoc nature of this analysis, additional caveats need consideration. The definition of “cognitive impairment” was based upon the MMSE score (21‐24), which is a relatively insensitive screening instrument for diagnosing mild cognitive impairment in PD.^43^ To put the MMSE score in context, the corresponding Montreal Cognitive Assessment scores for the cognitively impaired group would be 16 to 20. In addition, patients with more significant cognitive impairment (i.e., MMSE score < 21) were excluded. Finally, the 6‐week duration of the study prevents conclusions regarding long‐term tolerability and safety of PIM in patients with cognitive impairment or on cognitive‐enhancing medication.

In conclusion, we provide preliminary evidence that the effect of PIM may be robust in cognitively impaired PD patients and raise the possibility that concomitant use of cognitive‐enhancing medication may provide additional antipsychotic benefit. Future prospective randomized, controlled trials should evaluate the efficacy and tolerability of PIM in PD patients with a formal comorbid diagnosis of dementia, as well as in patients taking concomitant cognitive‐enhancing medications to evaluate possible synergistic effects.

## Author Roles

(1) Research Project: A. Conception, B. Organization, C. Execution; (2) Statistical Analysis: A. Design, B. Execution, C. Review and Critique; (3) Manuscript: A. Writing of the First Draft, B. Review and Critique.

A.J.E.: 1A, 1B, 2C, 3A, 3B

D.W.: 1A, 1B, 2C, 3A, 3B

M.T.G.: 1B, 1C, 2C, 3B

J.N.: 1B, 1C, 2C, 3B

N.L.: 2A, 3B

B.C.: 2A, 3B

J.A.V.: 2A, 3B

C.A.: 2A, 3B

D.F.: 2A, 3B

C.B.: 2A, 3B

S.A.F.: 2A, 3B

J.H.F.: 2A, 3B

A.E.L.: 2A, 3B

## Financial Disclosures

A.J.E. has received grant support from the NIH, Great Lakes Neurotechnologies, and the Michael J. Fox Foundation; personal compensation as a consultant/scientific advisory board member for AbbVie, TEVA, Impax, ACADIA, Acorda, Cynapsus/Sunovion, Lundbeck, and USWorldMeds; publishing royalties from Lippincott Williams & Wilkins, Cambridge University Press, and Springer; and honoraria from AbbVie, UCB, USWorldMeds, Lundbeck, ACADIA, the American Academy of Neurology, and the Movement Disorders Society. M.T.G., J.N., N.L., B.C., C.A., D.F., and C.B. are all employees of ACADIA Pharmaceuticals Inc. S.A.F. has received research grants from Ipsen, Medtronics, Teva, US World Meds, Sunovion Therapeutics, Solstice, Vaccinex, Voyager, Bristol‐Myers Squibb, Jazz Pharmaceuticals, CHDI Foundation, Michael J. Fox Foundation, and NIH; personal honoraria from Neurocrine, Lundbeck, Teva, Avanir, Sunovion, Biogen, Prexton Therapeutics, Adamas; and royalties from Demos, Blackwell Futura for textbooks, and UptoDate. J.H.F. is a paid consultant to ACADIA Pharmaceuticals; received royalties from Springer Press and Cambridge University Press; and received research funds from NIH and the Michael J. Fox Foundation. A.E.L. has served as an advisor for AbbVie, Acorda, Biogen, Bristol‐Myers Squibb, Janssen, Sun Pharma, Merck, and Corticobasal Degeneration Solutions; received honoraria from Sun Pharma, Medichem, Medtronic, AbbVie, and Sunovion; received grants from Brain Canada, Canadian Institutes of Health Research, Corticobasal Degeneration Solutions, Edmond J. Safra Philanthropic Foundation, Michael J. Fox Foundation, the Ontario Brain Institute, National Parkinson Foundation, Parkinson Society Canada, and W. Garfield Weston Foundation; and received publishing royalties from Elsevier, Saunders, Wiley‐Blackwell, Johns Hopkins Press, and Cambridge University Press. D.W. has received research funding or support from the Michael J. Fox Foundation for Parkinson's Research, National Institutes of Health (NINDS), Novartis Pharmaceuticals, Department of Veterans Affairs, Avid Radiopharmaceuticals, Alzheimer's Disease Cooperative Study, and the International Parkinson and Movement Disorder Society; honoraria from AbbVie, ACADIA, Biotie, Clintrex LLC, Janssen, Novartis, Pfizer, Teva Pharmaceuticals, UCB, and the CHDI Foundation; license fee payments from the University of Pennsylvania for the QUIP and QUIP‐RS; royalties from Wolters Kluweland; and fees for legal consultation for lawsuit related to antipsychotic prescribing in a patient with Parkinson's disease.

## Supporting information


**FIG. S1.** Percent of patients taking cognitive‐enhancing medications by baseline MMSE score.

**FIG. S2.** CGI‐I score in patients taking concomitant (A), any cholinesterase inhibitor (B), and memantine (C). LSM, least squares mean; SE, standard error; PIM, pimavanserin; PBO, placebo.
