# Supplementary figures and images for: Pimavanserin for Parkinson's Disease psychosis: Effects stratified by baseline cognition and use of cognitive‐enhancing medications
Source: Mov Disord. 2018 Nov 2;33(11):1769–76. doi: 10.1002/mds.27488 (PMC6261678; doi:10.1002/mds.27488)

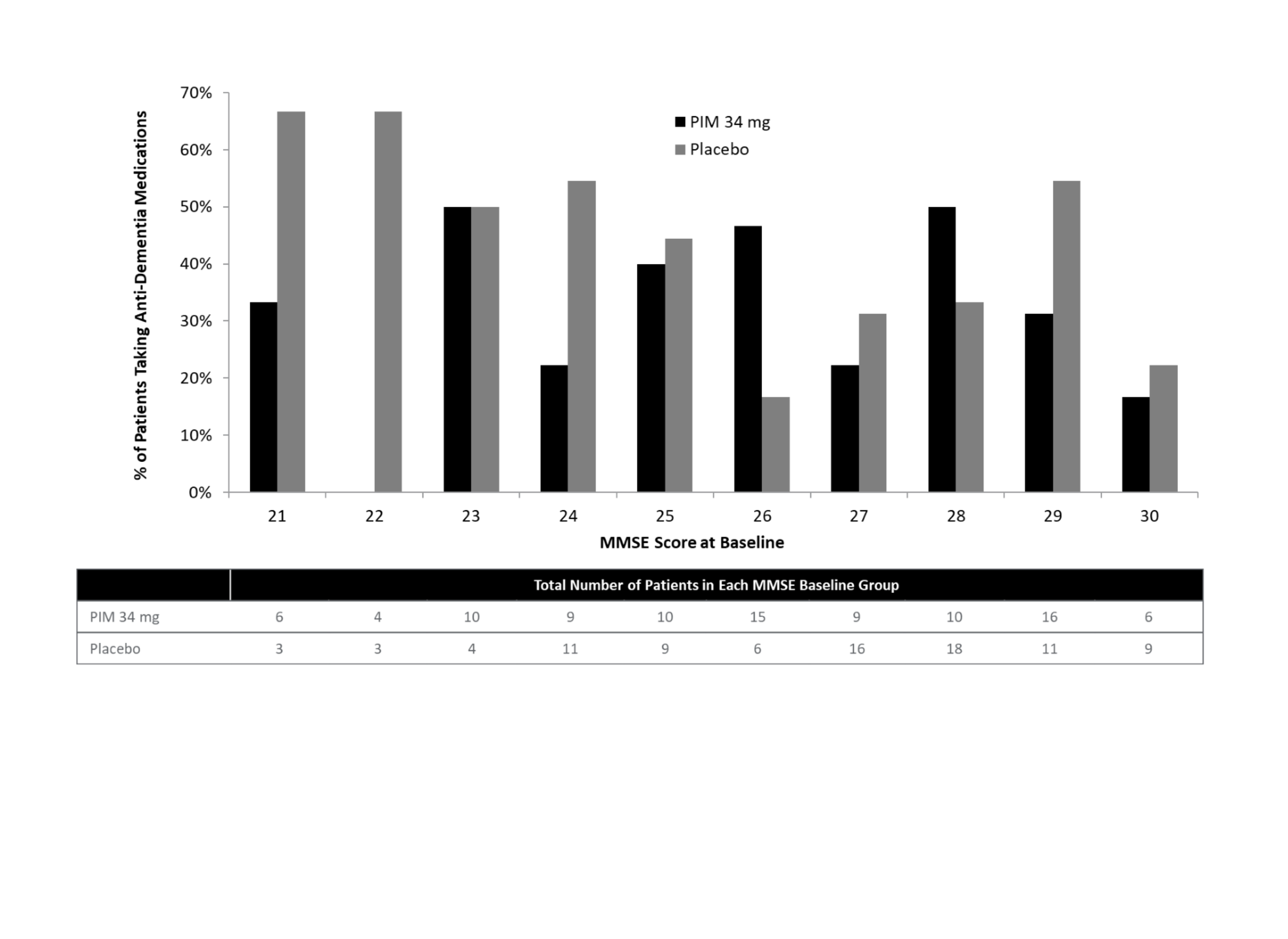

Supplement: Supplementary file 1 — FIG. S1. Percent of patients taking cognitive‐enhancing medications by baseline MMSE score. [file MDS-33-1769-s001.TIF]

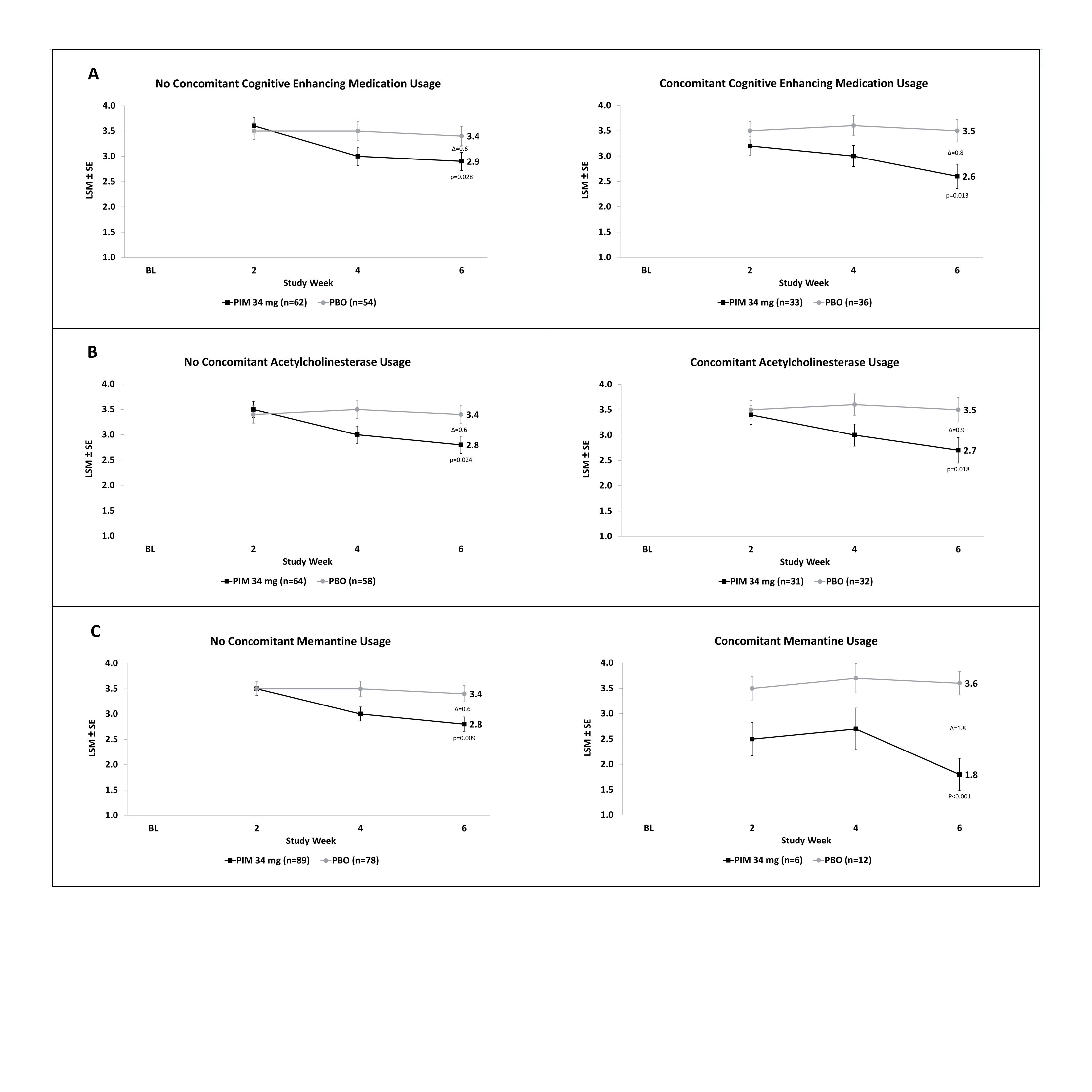

Supplement: Supplementary file 2 — FIG. S2. CGI‐I score in patients taking concomitant (A), any cholinesterase inhibitor (B), and memantine (C). LSM, least squares mean; SE, standard error; PIM, pimavanserin; PBO, placebo. [file MDS-33-1769-s002.tif]
